# Herpes zoster surveillance using electronic databases in the Valencian Community (Spain)

**DOI:** 10.1186/1471-2334-13-463

**Published:** 2013-10-05

**Authors:** Nuria Morant-Talamante, Javier Diez-Domingo, Sergio Martínez-Úbeda, Joan Puig-Barberá, Sara Alemán-Sánchez, Lina Pérez-Breva

**Affiliations:** 1 Vaccine Research Area, Foundation for the Promotion of Health and Biomedical Research in the Valencian Region FISABIO - Public Health, Valencia, Spain

**Keywords:** Herpes Zoster, Epidemiology, Electronic health record, Spain

## Abstract

**Background:**

Epidemiologic data of Herpes Zoster (HZ) disease in Spain are scarce. The objective of this study was to assess the epidemiology of HZ in the Valencian Community (Spain), using outpatient and hospital electronic health databases.

**Methods:**

Data from 2007 to 2010 was collected from computerized health databases of a population of around 5 million inhabitants. Diagnoses were recorded by physicians using the International Classification of Diseases, 9th Revision, Clinical Modification (ICD-9-CM). A sample of medical records under different criteria was reviewed by a general practitioner, to assess the reliability of codification.

**Results:**

The average annual incidence of HZ was 4.60 per 1000 persons-year (PY) for all ages (95% CI: 4.57-4.63), is more frequent in women [5.32/1000PY (95% CI: 5.28-5.37)] and is strongly age-related, with a peak incidence at 70-79 years. A total of 7.16/1000 cases of HZ required hospitalization.

**Conclusions:**

Electronic health database used in the Valencian Community is a reliable electronic surveillance tool for HZ disease and will be useful to define trends in disease burden before and after HZ vaccine introduction.

## Background

Herpes Zoster (HZ; shingles) results from the reactivation of Varicella-Zoster Virus (VZV) that has been latent in the spinal and cranial sensory ganglia after primary infection with varicella (chickenpox), usually during childhood [1]. A vesicular skin rash in the affected dermatome, commonly accompanied by acute pain, characterizes the acute phase of HZ disease.

Approximately 14% of all patients with shingles will develop complications [2]. The most common and weakening is postherpetic neuralgia (PHN), defined by most investigators as pain being present at 90 days after the rash appears [1,2]. Both the acute pain associated with HZ and PHN have a negative impact on health-related quality of life, interfering significantly with physical, emotional, and social functioning [3-6], and quantitatively similar to congestive heart failure, severe depression, acute myocardial infarction, or uncontrolled diabetes [7].

The risk of HZ and its complications increases with advancing age, being more manifest in persons over 50-60 years of age [1,2,8,9]. Among individuals who reach age 85 years of age, approximately 50% will have experienced at least one episode of HZ [10]. The incidence of acute HZ in the European general population ranges between 2.0-4.8 per 1000 persons-year (PY) for all ages, and increases after age 50 years to around 7-8/1000 PY [11,12]. Over the coming decades, Europe’s demographic patterns will change dramatically, and it is expected that our populations will become older than ever before [13]. This suggests that the incidence of HZ could vary in relation to an older population, possibly as a result of immunosenescence [14].

Several studies support the hypothesis that exposure to childhood varicella reduces the risk of developing HZ by boosting specific immunity to VZV [15-19]. Based on this hypothesis, mathematical models suggest that a successful childhood vaccination program may decrease the circulation of wild-type VZV, and therefore the stimulation of cellular immunity that could prevent the occurrence of shingles in the adult population [20-23]. This could have significant public health consequences, if a universal mass VZV vaccination program is implemented in childhood. Although this issue is still controversial and there is no firm evidence that this increase will occur [24-26], monitoring of disease trends over time will help understand the impact of different factors upon the incidence of HZ. In the Valencian Community (Spain), varicella vaccination is recommended and funded at 12 years of age in those subjects who have not been in clear contact with the virus. This helps avoid severe cases in adults with no impact upon circulation of the virus. About 30% of toddlers also receive the vaccine after their pediatrician’s recommendation [27]. With this low coverage figure, the virus is circulating within the population and no effect upon HZ is expected.

The efficacy of a live attenuated zoster vaccine, Zostavax®, in preventing HZ and PHN has been tested in adults ≥ 60 years of age, yielding efficacy rates of 51% and 67%, respectively [28], and of 70% for incident HZ in subjects aged 50–59 years old [29]. In a recent population-based study of 766,330 individuals aged ≥ 65 years, the effectiveness was seen to be 48% [30]. This vaccine is generally well tolerated, has been licensed in the European Union in 2006 for people aged ≥50 years [31], and is expected to be widely used in Spain over the next few years. Another adjuvanted vaccine is under development [32-34].

In Spain, HZ is presently not a notifiable disease, and epidemiological data are scarce. A recent prospective study in the Valencian Community (Spain) showed an HZ incidence of 4.1 per 1000 persons > 14 years of age during 2007 [35,36]. Reliable epidemiological data are needed before any universal recommendation of HZ vaccines can be made, in order to assess the impact of HZ vaccination. The purpose of this study was to explore the epidemiology of HZ in the Valencian Community during a four-year period (January 2007 to December 2010), and to estimate the reliability of the regional electronic medical database for epidemiological studies, with a view to creating surveillance tools for future and efficient assessments of HZ incidence in the post-HZ vaccine era.

## Methods

### Setting and study population

The Valencian Community, in the east of Spain, has a population of 5,117,190 inhabitants (2011) [37], and over 98% are covered by the national public health system (NHS) [38]. Primary care visits and hospitalizations are recorded in clinical databases. Using these, we sought cases of HZ of all ages and both sexes attended in the NHS from 1 January 2007 to 20 December 2010. From each HZ case we obtained all medical visits, prescriptions and demographic data.

### Abucasis electronic healthcare database

The Abucasis electronic medical database was implemented in the Valencian Community for outpatient and primary care settings in 2006, and offers the possibility of linking patient care and public health [39]. From 2006 to 2010 (when the whole health system was computerized), the percentage of the population included in Abucasis increased from 73.1% in 2007 to 88.8% in 2008 and 95.7% in 2009 (Abucasis managers, personal communication). Abucasis contains an ambulatory information system called SIA, which registers any medical contact (visit), and the attending physician uses a drop-down menu with the International Classification of Diseases, 9th Revision, Clinical Modification (ICD-9-CM) to record diagnoses. Abucasis also links other databases: Care Provision Management (GAIA), which is the drug information system available to the different professionals involved in prescription and dispensation, addressed from the Department of Health; and the Vaccine Information System (RVN) [40]. Other databases used in this study, such as the Hospital Data Surveillance System (CMBD), are described elsewhere [41] and can be linked through a unique personal identification number (SIP), for the collection of demographic data.

### Case definition

For the identification of incident cases of HZ, we searched SIA for any subject with first appearance of an HZ-related ICD-9-CM code (all ICD-9-CM 053 codes: HZ or HZ related complications), and the CMBD database for an HZ diagnosis in any position (1st to 9th). GAIA was searched for information on all subjects who were prescribed antiviral drugs (acyclovir, famcyclovir or valacyclovir) at doses only licensed for use in HZ by the Spanish Medicines Agency (AEMPS).

Any outpatient medical contact or visit, hospital admission or electronic prescription related to HZ was considered as a medical encounter. In order to avoid an overestimation of results, and in an attempt to identify recurrent HZ episodes, each medical encounter not preceded by another encounter in the last six months, and succeeded by another encounter in the following three months was considered as a recurrent HZ case.

To assess data quality, a total of 550 medical records were reviewed by a physician (NMT) in order to assess coding reliability and the accuracy of the filters used. These medical records were randomly selected after request of the following criteria: definition of incident HZ case and recurrent HZ case (300 records reviewed), prescription of specific topical and oral HZ antiviral drugs without a specific HZ ICD-9-CM code (100 records reviewed), discordance of the narrative diagnostic description with the assigned ICD-9-CM HZ code (100 records reviewed), and hospitalizations with an HZ diagnosis not appearing in the Abucasis database (50 records reviewed). In order to determine recurrence, each medical contact in the Abucasis database from 1 June 2006 to 31 March 2011 was evaluated. Confirmation of an HZ diagnosis required an HZ ICD-9-CM code in addition to application of at least one of the following criteria:

1. Detailed physician description of characteristic HZ skin lesions

2. Prescription of antiviral drugs

3. Temporary disability under HZ diagnosis

4. Referral for specialist assessment due to HZ disease, without a subsequent diagnostic exclusion of HZ diagnosis

Our data from 2007 were graphed together with the results obtained in a regional prospective cohort study in patients aged > 14 years and carried out in 25 primary care settings in the Valencian Community during the same year [35,36].

All databases were merged using the database manager MySQL 5.1. The random selection for quality control of coding was performed using its “rand” function. Analyses were performed using MySQL and Epidat 3.1. We calculated annual incidence rates of HZ by dividing the number of cases by the persons registered in SIP in each year. The exact 95% confidence interval (CI) for the incidence rates was calculated on the basis of a normal distribution. The Risk Ratio was calculated to assess differences between males and females.

### Ethical considerations

The study protocol abided with the principles of the Declaration of Helsinki and was approved by the Public Health Ethics Committee of Valencia. Waiver of informed consent was accepted for medical history review.

## Results

### HZ case confirmation

We identified a total of 85,586 persons in SIA and/or CMBD with a first diagnosis of HZ. Our criteria of recurrence were met by 3300 of them. After chart review, the positive predictive value (PPV) for HZ case definition was 92.7% (95% CI 89.1-95.4), and for recurrent HZ cases was 55.1% (95% CI 47.0-63.0). Due to the low PPV of the recurrence filter, only the incident HZ cases were included in our estimations.

The PPV for HZ diagnosed only by high-dose antiviral prescription was 26% (95% CI: 17.7-35.7), therefore, no case identified only by the prescription of antivirals was considered as an HZ case.

### Epidemiological analysis

#### HZ incidence

**Figure 1:**
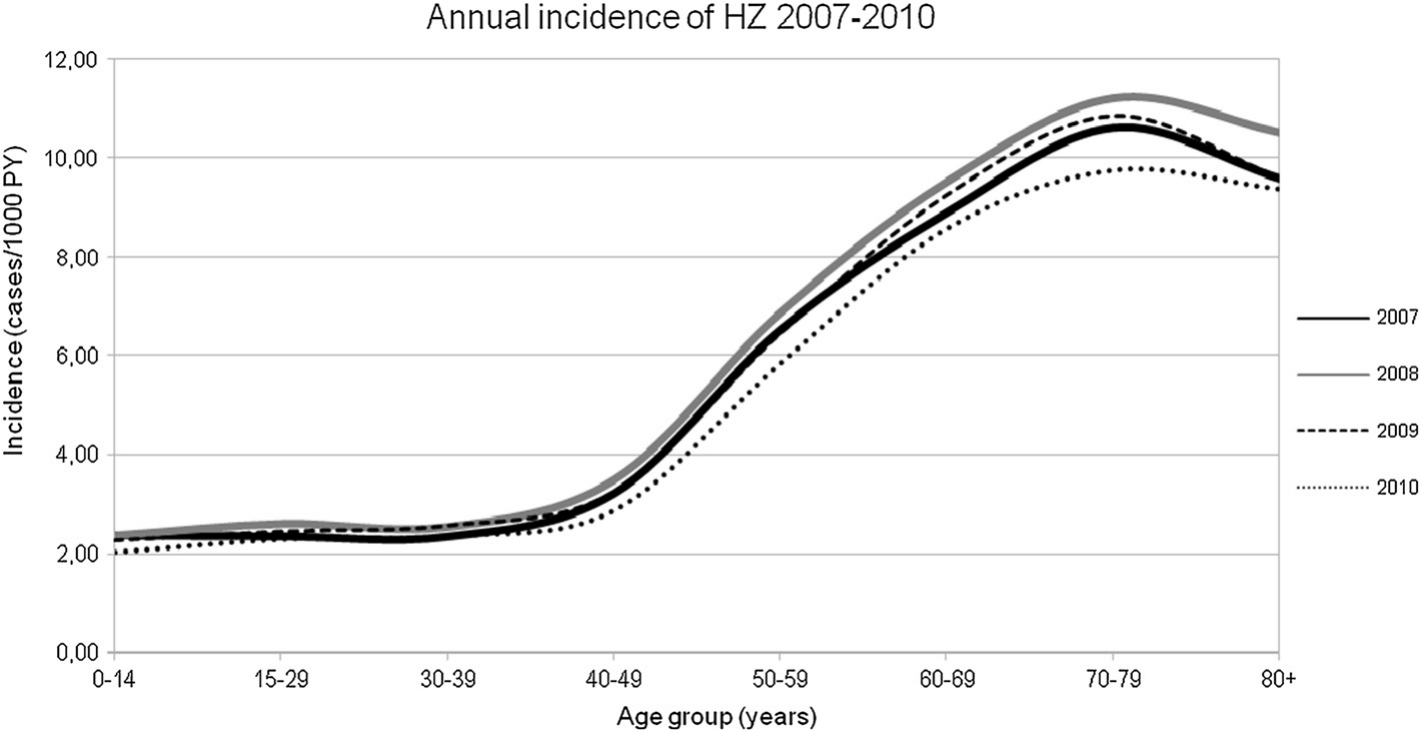
Incidence of HZ / 1000 person-years by age-groups, Valencian Community, Spain,years 2007-2010.

Over the four-year study period, we identified 85,586 persons with incident cases of HZ requiring medical care, which correspond to an incidence in all age groups of 4.60/1000 PY (95% CI: 4.57-4.63). The figures remained rather constant over the years: 4.58/1000 PY (95% CI: 4.51-4.65) in 2007, 4.89 (95% CI: 4.83-4.95) in 2008, 4.67 (95% CI: 4.61-4.73) in 2009, and 4.29 (95% CI: 4.24-4.35) in 2010 (Figure 1).

HZ incidence rates were strongly aged-related, and more than half of the cases involved patients over 50 years of age (63.18%) (Table 1). The incidence in the non-pediatric age group (≥ 15 years) was 5.02/1000 PY (95% CI: 4.99-5.06), while in the target population for HZ vaccine (adults aged ≥50 years) the incidence was 8.60/1000 PY (95% CI: 8.53-8.67). This trend was maintained each year during the whole study period. An incidence peak was observed in those aged 70-79 years, with a maximum of 11.55/1000 PY (95% CI: 11.32-11.79) in women and 9.41/1000 PY (95% CI: 9.18-9.65) in men. A drop in incidence in the older age group occurred in the primary care database but was not seen in the hospitalizations (Table 2).

**Table 1:** Annual incidence rate (2007-2010) for HZ per 1000 PY (adjusted population*) per age-group in the Valencian Community (Spain)

	**2007**		**2008**		**2009**		**2010**		**2007-2010 MALES**	**2007-2010 FEMALES**
**Age group (years)**	**Cases (Population)**	**Incidence (95% CI)**	**Cases (Population)**	**Incidence (95% CI)**	**Cases (Population)**	**Incidence (95% CI)**	**Cases (Population)**	**Incidence (95% CI)**	**Incidence (95% CI)**	**Incidence (95% CI)**
0–14	1324 (561405)	2.36 (2.23–2.49)	1638 (690421)	2.37 (2.26–2.49)	1715 (762629)	2.25(2.14–2.36)	1610 (800131)	2.01(1.91–2.11)	2,12 (2.04–2.19)	2.36 (2.27–2.44)
15–29	1702 (727512)	2.34 (2.23–2.45)	2246 (859724)	2.61 (2.50–2.72)	2219 (912691)	2.43(2.33–2.53)	2077 (914068)	2.27(2.17–2.37)	2,26 (2.19–2.34)	2,57 (2.49–2.65)
30–39	1548 (663307)	2.33 (2.22–2.45)	2051 (804937)	2.55 (2.44–2.66)	2271 (890097)	2.55 (2.45–2.66)	2156 (923521)	2.33 (2.24–2.4)	2,19 (2.12–2.26)	2.72 (2.64–2.80)
40–49	1796 (558784)	3.21 (3.07–3.36)	2401 (685727)	3.50 (3.36–3.64)	2460 (761831)	3.23 (3.10–3.36)	2299 (804414)	2.86 (2.74–2.97)	2,68 (2.59–2.76)	3,72 (3.62–3.82)
50–59	2782 (426382)	6.52 (6.28–6.77)	3586 (521416)	6.88 (6.65–7.10)	3732 (579549)	6.44 (6.23–6.65)	3630 (621615)	5.84 (5.65–6.03)	4,85 (4.72–4.99)	7,87 (7.70–8.04)
60–69	3200 (359492)	8.90 (8.59–9.21)	4145 (435089)	9.53 (9.24–9.82)	4534 (489996)	9.25 (8.99–9.52)	4414 (514783)	8.57 (8.32–8.83)	7.73 (7.54–7.91)	10.26 (10.05–10.46)
70–79	3235 (304607)	10.62 (10.26–10.98)	4026 (359151)	11.21 (10,87–11.55)	4188 (385600)	10.86 (10.53–11.19)	3939 (403616)	9.76 (9.46–10.06)	9.41 (9.18–9.65)	11.55 (11.32–11.79)
≥ 80	1718 (179060)	9.59 (9.14–10.05)	2265 (215358)	10.52 (10.09–10.95)	2331 (242269)	9.62 (9.23–10.01)	2348 (250391)	9.38 (9.00–9.75)	9.55 (9.21–9.89)	9.88 (9.27–10.14)
Total	17305 (3780550)	4.58 (4.51–4.65)	22358 (4571822)	4.89 (4.83–4.95)	23450 (5024661)	4.67 (4.61–4.73)	22473 (5232539)	4.29 (4.24–4.35)	3.86 (3.82–3.90)	5.32 (5.26–5.37)

*Adjusted population: Correction factor 73.09% (2007), 88.75% (2008), 95.72% (2009), 100% (2010).

**Table 2:** Hospitalizations due to Herpes Zoster by age-groups in the Valencian Community (Spain), 2007-2010

**Age group (years)**	**Population**	**Cases**	Incidence *	**95% CI**	**Total HZ cases in the population**	Hospitalization rates**
0–14	3,142,807	40	1.27	0.88 – 1.67	6287	6.36
15–29	3,831,527	22	0.57	0.33 – 0.81	8244	2.67
30–39	3,667,802	47	1.28	0.92 – 1.65	8026	5.86
40–49	3,137,382	49	1.56	1.12 – 2.00	8956	5.47
50–59	2,397,883	52	2.17	1.58 – 2.76	13730	3.79
60–69	2,008,718	91	4.53	3.60 – 5.46	16293	5.60
70–79	1,627,844	156	9.58	8.08 – 11.09	15388	10.14
80+	991,106	156	15.74	13.27 – 18.21	8662	18.01
Total	20,805,069	613	2.95	2.71 – 3.18	85586	7.16

*Cases per 100,000 person-years.

**Hospitalization rates (number of hospitalizations per 1000 cases of HZ).

HZ was less common among men [3.86/1000 PY (95% CI: 3.82-3.90)] than women [5.32/1000PY (95% CI: 5.28-5.37)] in the total population, with a RR 0.73 (95 CI: 0.72-0.74) (Table 1).

A total of 1458 hospitalizations with HZ related ICD-9-CM codes occurred during the study period (an annual average of 365 admissions). Of these, 683 were men (46.8%) and 775 women (53.2%). The mean age was 67.5 years (range 0-100). Characteristics referred to hospitalized population and hospitalization rates are shown in Tables 2 and 3.

**Table 3:** Characteristics of hospitalizations due to Herpes Zoster in the Valencian Community (Spain) 2007-2010

**Hospital admissions**	**2007**	**2008**	**2009**	**2010**	**Total**
***Discharges with 1st-9th listed HZ related ICD-9-CM code***					
Number of admissions	325	395	334	401	1458
Median age (years) (min-max)	66.67 (1–95)	67.64 (0–97)	66.47 (0–100)	68.94 (1–99)	67.51 (0–100)
Men/Women	155/173	172/223	152/182	201/200	680/778
Sex ratio (M/W)	0.89	0.77	0.83	1.01	0.87
Average length of stay (days) (SD)	10.56 (11.43)	10.12 (12.01)	9.82 (10.63)	8.65 (8.29)	9.75 (10.67)
***Discharges with 1st-2nd listed HZ related ICD-9-CM code***					
Number of admissions	135	153	136	189	613
Median age (years) (min-max)	60.19 (1–95)	62.49 (0–93)	60.19 (0–97)	65.50 (1–97)	62.44 (0–97)
Men/Women	61/74	73/80	63/73	98/91	295/318
Sex ratio (M/W)	0.82	0.91	0.86	1.08	0.93
Average length of stay (days) (SD)	7.73 (6.78)	7.98 (7.69)	7.15 (5.93)	7.47 (5.96)	7.59 (6.61)

HZ was the first coded diagnosis in 383 cases (26.2% of all hospitalizations). For these cases, secondary codes were hypertension (7.8%), hematopoietic malignancies (3.4%), HIV infection (2.6%), diabetes mellitus (2.3%) and urinary tract infection (1.3%). In 5.7% of the cases, HZ was the only diagnostic code. HZ was the second listed diagnosis in 250 admissions (17.1%). For these cases, first coded diagnoses were respiratory tract infectious diseases (8%), meningitis (6%), HIV infection (6%) and chronic obstructive pulmonary disease (2%).

The most frequently coded HZ diagnosis was ICD-9-CM 053.9 (HZ without complications) (Table 4). A total of 679 patients admitted to hospital with HZ as first listed diagnosis were not coded as a HZ case in SIA. Chart review of 50 of these patients showed the reasons for not coding to be death, change of address, lack of follow-up, or no HZ ICD-9-CM codification but mentioning hospitalization under other ICD-9-CM code.

**Table 4:** Number of Herpes Zoster - related medical encounters (visits) and proportion of HZ diagnoses (ICD-9-CM codes), 2007-2010

**ICD-9-CM codes (Description)**	**Number of medical visits (proportion %)**		
	**Hospital admissions**		Outpatient^*^
	***ICD-9-CM1*st*-9*th**	***ICD-9-CM1*st*-2*nd**	
053.9 (Herpes zoster without complications)	812 (55.50)	328 (53.52)	70816 (36.93)
0.53.19 (Herpes zoster with other nervous system complications)	330 (22.63	94 (14.85)	3326 (1.73)
053.79 (Herpes zoster with other specified complications)	61 (4.17)	46 (7.27)	532 (0.28)
053.20 (Herpes zoster with ophthalmic complications)	51 (3.49)	28 (4.42)	7313 (0.38)
053.29 (Herpes zoster with other ophthalmic complications)	50 (3.42)	28 (4.42)	312 (0.16)
053.12 (Postherpetic trigeminal neuralgia)	45 (3.08)	20 (3.16)	4729 (2.47)
053.13 (Postherpetic polyneuropathy)	32 (2.19)	10 (1.58)	1821 (0.95)
053.11 (Geniculate herpes zoster)	29 (1.98)	19 (3.02)	496 (0.26)
053.8 (Herpes zoster with unspecified complications)	27 (1.85)	21 (3.43)	663 (0.35)
053 (Herpes Zoster without specification)	0	0	94950 (49.52)
Other	56 (3.82)	39 (6.16)	6778 (3.54)
**Total (medical visits under HZ ICD-9-CM codes / number of patients)**	**1463/1458**	**633/613**	**191736 (85586)**

*Outpatient:* * includes HZ ICD-9-CM codifications (053.0-053.9) for all medical visits.

**Figure 2:**
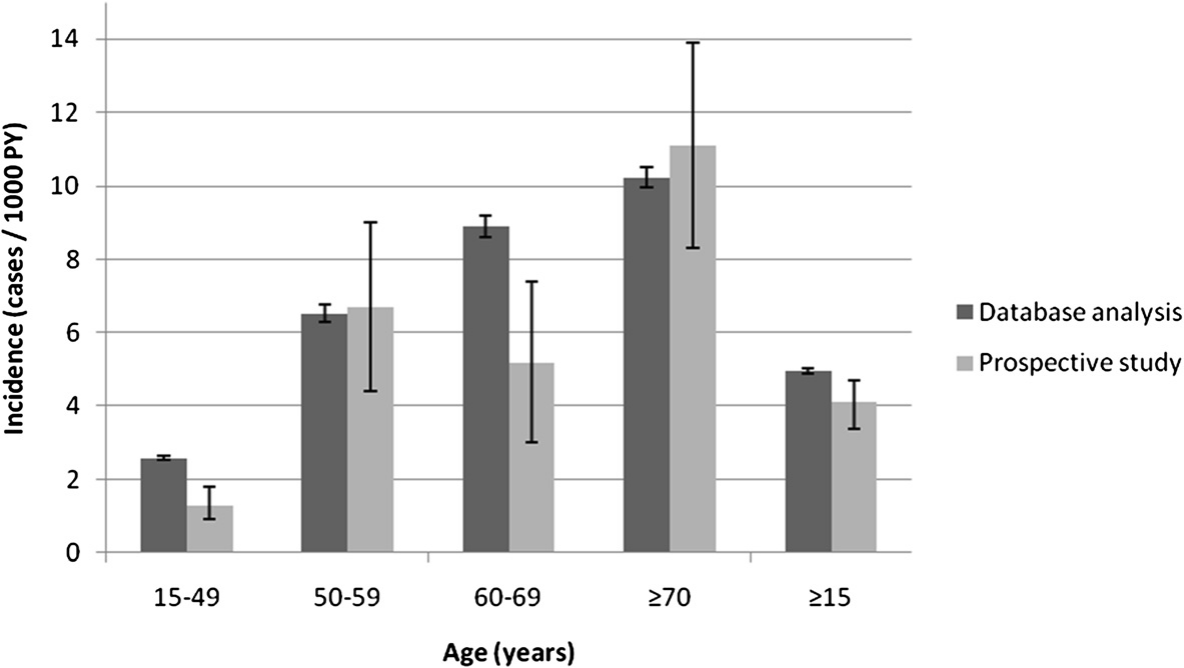
**Description of the incidence of HZ per 1000 person-year by age (95% CI) in this study and in a prospective study in Valencia **[35]**.**

Figure 2 depicts the age incidence in this study and in the prospective study [35]. In both studies the annual incidence was higher in females. The prospective study showed an incidence of 4.5/1000 PY (95% CI: 3.5-5.4) in females and 2.7/1000 PY (95% CI: 1.9-3.5) in males, while the electronic database showed an incidence of 5.32 (5.26-5.37) per 1000 PY for women and 3.86 (3.82-3.90) per 1000 PY for men (Table 1).

## Discussion

In this study we used medical electronic databases from a region of Spain in order to reliably estimate HZ incidence. Our results showed an HZ incidence of 4.60/1000 PY, with a strong correlation to age, and with a drop in incidence in patients ≥ 80 years of age, which was not observed in hospitalizations. Incidence rates were higher among women in most age groups, with an overall figure of 3.86 in males and 5.32 PER 1000 PY in females.

Epidemiological studies on HZ in Spain are scarce and are needed for future estimations of disease burden [35,36,42,43]. Our study used health databases that cover almost the entire population living in the Valencian Community, including hospitalizations and ambulatory patients. To the best of our knowledge, there is only one study of similar characteristics in Spain, conducted in Navarra (a northern region of the country) before systematic varicella vaccination [44].

One of the limitations of the study is that the Abucasis database was not developed for medical billing purposes, and this may imply the obtainment of less detailed information. Although ICD-9-CM codes are routinely used, general practitioners (GP) may be unaware of its importance. To overcome this, codification is performed in simple way – the system presenting a list of possible ICD-9-CM codes after the diagnosis is written. In the case of Zoster, which is a common diagnosis, with no synonyms, ICD-9 coding is relatively easy. On the other hand, trained personnel codify CMBD. In order to assess the reliability of HZ codification, a random review of medical records showed good matching between ICD-9-CM coding and a real episode of HZ. A total of 7.7% of the reviewed notes with the diagnosis of Herpes Zoster did not meet the full requirements for being considered a case. Most of these situations were due to a lack of description of the lesions. We considered that most of these cases could be HZ, but the GP did not provide sufficient written details, possibly because of the great care burden found in primary care, which prevents entering detailed information in medical notes. This consequently may have understated HZ case confirmation.

For recurrent cases our temporal filters were not wholly suitable, and therefore the system did not allow for analysis of recurrent cases.

Another limitation is that emergency room visits and private medical visits were not included in any of the databases. Since the medication usually prescribed for HZ is expensive and is not subsidized in these situations, we assumed that only a minority of patients would not seek public medical care in order to receive subsidized drugs. As a population database study, it is possible that some individuals did not seek medical care. Another limitation to be taken into account is that we only included clinical diagnoses of HZ, since laboratory confirmation of HZ is rarely used in normal clinical practice.

Due to bureaucratic problems, SIA data for the year 2010 were given until 20 December. We assume that the remaining 11 days would have no significant impact upon our estimations.

On comparing results corresponding to 2007 from the Abucasis database and a prospective study carried out in a similar population (>14 years) [35,36], the incidence figures were found to be similar, being slightly higher when using the electronic database (5.0/1000 PY versus 4.1/1000 PY). These differences mainly occurred in two age groups (15-49 and 60-69 years), and as the authors point out [35], this may reflect their low precision. The study conducted in Navarra [42] showed a mean HZ incidence of 4.25/1000 PY during 2005-2006.

The number of hospitalizations in our study was lower than in similar retrospective Spanish studies during the previous study period [41,42]. In one of these studies [41], differences were found among the Spanish autonomous communities, with lower incidence rates in the Valencian Community. To confirm the reliability of our data, we compared them with the national data for the same study period (data not shown), and found both figures to be the same, with a decrease in the incidence of HZ hospitalization over time, possibly due to changes in admission criteria, to greater awareness among physicians of the need for early antiviral treatment, or to changes in coding practices. However, similar hospitalizations rates were found (2.69/100,000 PY) in another Spanish study using the CMBD database for the study period 1997-2004 [45].

As in other epidemiological studies, the incidence of HZ is strongly sex- and age-related, with higher incidences in women over 50 years. Our incidence was slightly higher than in other European studies using electronic databases. In France, the yearly HZ incidence rate for all ages averaged 3.82/1000 PY in the study period 2005-2008 [46], while in Italy a retrospective study showed an incidence of 4.31/1000 PY for population aged 15 years or older during the three-year study period (2003-2005) [47]. Another study conducted in the Netherlands during 1994-1999, calculated an HZ incidence of 3.4/1000 PY [48]. A similar German study performed in an older population (≥ 50 years) reported higher figures (9.60/1000 PY during 2007-2008) [49]. We found similar rates in the United States: 4.4/1000 PY for all ages during 2006 [50]. Apart from the fact that the results of different descriptive epidemiological studies are highly dependent upon the methodology used, some of these studies included emergency room visits, and some countries use ICD-9-CM for billing purposes, which would result in variable incidence rates.

Higher incidence rates were found in the placebo controls of clinical trials in the ≥ 60 years age group, specifically 13.00/1000 PY [50,51] or 11.12/1000 PY [28], which indicates that an active search of HZ cases considerably increases their incidence.

Our incidence peaked at 70-79 years of age and decreased thereafter, especially in the ≥95 years age group (data not shown). This drop in incidence was not seen in hospitalizations (Table 3). There are several explanations for this. Firstly, there could be a gradually reduced risk of HZ, explained by the hypothesis that exposure to VZV provides the host with progressive immunity to VZV reactivation [52,53]. Secondly, large proportions of subjects in this age group have disabilities or walking difficulties, and consequently are usually visited by family physicians at home. These visits are commonly not recorded in the Abucasis database, and medical prescriptions are handwritten. On the other hand, many of these patients live with relatives and usually move to other provinces outside our study area during part of the year, without unsubscribing from the Abucasis database; a potential HZ case therefore could have been registered elsewhere and not be counted as an incident case.

HZ incidence is age-related. This correlation could be explained by the progressive decline in VZV cell-mediated immunity related to aging. The incidence should increase in a progressively aging population, which would imply a greater burden and cost of disease. Determinants of direct costs of an HZ episode are usually related to the prescription of antiviral drugs and repetitive primary care visits in the case of PHN. Direct outpatient costs in several European countries average between 72.05 € and 247 € [36,54-56].

## Conclusions

Our study confirms electronic databases as a reliable epidemiological tool for estimating the incidence of HZ disease. They provide an important source of information on the incidence of HZ, which can be useful to define trends in disease burden before and after HZ vaccine introduction.

## Abbreviations

HZ: Herpes Zoster; PHN: Postherpetic neuralgia; PY: Persons-year; NHS: National health system; SIA: Sistema de información ambulatoria (Ambulatory Information System); ICD-9-CM: International classification of diseases 9th revision, clinical modification; CMBD: Conjunto Mínimo Básico de Datos (Hospital Data Surveillance System); PPV: Positive predictive value; SPS: Shingles prevention study; VZV: Varicella-zoster virus; GP: General practitioner.

## Competing interests

JDD Is acting as national coordinator and principal investigators for clinical studies and receiving funding from non-commercial funding bodies as well as commercial sponsors (Novartis Vaccines, GlaxoSmithKline, Baxter, Sanofi Pasteur MSD, MedImmune, and Pfizer Vaccines) conducted on behalf of CSISP-FISABIO. He served as a as a board member for GSK, and received payment for lectures from SP-MSD, Novartis and Baxter that included support for travel and accommodation for meetings.

JPB Is acting as national coordinator and principal investigators for clinical studies and receiving funding from non-commercial funding bodies as well as commercial sponsors (Novartis Vaccines, GlaxoSmithKline and Sanofi-Pasteur) conducted on behalf of CSISP-FISABIO. He served as a as a board member for GSK, and received payment for lectures that included support for travel and accommodation for meetings.

Other authors declare no conflict of interest.

## Authors’ contributions

NMT, JDD and JPB designed the study. SMU analyzed the data and performed the statistical analysis. NMT wrote the manuscript and reviewed clinical records. JDD coordinated the study. SAS, LPB, JDD and JPB provided valuable insight for revising the manuscript. All authors read and approved the final manuscript.

## Pre-publication history

The pre-publication history for this paper can be accessed here:

http://www.biomedcentral.com/1471-2334/13/463/prepub
